# Palatal development of preterm and low birthweight infants compared to term infants – What do we know? Part 2: The palate of the preterm/low birthweight infant

**DOI:** 10.1186/1746-160X-1-9

**Published:** 2005-10-28

**Authors:** Ariane Hohoff, Heike Rabe, Ulrike Ehmer, Erik Harms

**Affiliations:** 1 Poliklinik für Kieferorthopädie, Universitätsklinikum, Westfälische Wilhelms-Universität, Münster, Germany; 2 Department of Neonatology, Brighton & Sussex University Hospitals, UK; 3 Klinik für Kinderheilkunde, Division of Neonatology, Universitätsklinikum, Westfälische Wilhelms-Universität, Münster, Germany

## Abstract

**Background:**

Well-designed clinical studies on the palatal development in preterm and low birthweight infants are desirable because the literature is characterized by contradictory results. It could be shown that knowledge about 'normal' palatal development is still weak as well (Part 1). The objective of this review is therefore to contribute a fundamental analysis of methodologies, confounding factors, and outcomes of studies on palatal development in preterm and low birthweight infants.

**Methods:**

An electronic literature search as well as hand searches were performed based on Cochrane search strategies including sources of more than a century in English, German, and French. Original data were recalculated from studies which primarily dealt with both preterm and term infants. The extracted data, especially those from non-English paper sources, were provided unfiltered for comparison.

**Results:**

Seventy-eight out of 155 included articles were analyzed for palatal morphology of preterm infants. Intubation, feeding tubes, feeding mode, tube characteristics, restriction of oral functions, kind of diet, cranial form and birthweight were seen as causes contributing to altered palatal morphology. Changes associated with intubation concern length, depth, width, asymmetry, crossbite, and contour of the palate. The phenomenon 'grooving' has also been described as a complication associated with oral intubation. However, this phenomenon suffers from lack of a clear-cut definition. Head flattening, pressure from the oral tube, pathologic or impaired tongue function, and broadening of the alveolar ridges adjacent to the tube have been raised as causes of 'grooving'. Metrically, the palates of intubated preterm infants remain narrower, which has been examined up to the age of the late mixed dentition.

**Conclusion:**

There is no evidence that would justify the exclusion of any of the raised causes contributing to palatal alteration. Thus, early orthodontic and logopedic control of formerly orally intubated preterm infants is recommended, as opposed to non-intubated infants. From the orthodontic point of view, nasal intubation should be favored. The role that palatal protection plates and pressure-dispersing pads for the head have in palatal development remains unclear.

## Background

Compared to their term counterparts, prematurely born babies are at risk for postnatal growth and development defects. The general objective of neonatal care of premature infants is to support life and ensure a growth rate sufficient to fulfill the individual's genetic potential. Reaching this goal has undergone a dramatic improvement in the last two decades. Therefore, research into the development of these patients can and must now be extended to other areas, such as their physical and cognitive development. The morbidity potential associated with prematurity needs to be investigated to establish preventive measures. The orofacial region plays an important role in the infant's development. Premature babies must develop the skills needed to begin oral feeding as they reach an age that supports coordination of breathing and swallowing. This normality of oral functions, including nose breathing, induces adequate development of the whole orofacial region. During the time period from initiation of oral functions to full oral feeding in neonatal care, a complex interplay of various internal and external factors exerts an influence that may affect palatal development and lead therefore to a higher risk for malocclusions, facial asymmetries, and other late consequences. The evidence on these possible consequences is weak and the results of one century of research on palatal development are still controversial. This applies also to the knowledge gained on 'normal' palatal development of term babies (Part 1) which is a precondition to recognize abnormalities in the preterm infant's palate.

The objective of Part 2 of this review is therefore to contribute a fundamental analysis of methodologies, confounding factors, and outcomes of studies on palatal development of preterm and low birthweight infants.

## Methods

The search strategy, the surveyed medical databases and sources of hand searches, and the assessment of included studies are described in Part 1. Exclusion criteria and excluded articles are listed in detail in Table 1 of Part 1 (see Additional file 1 of Part 1). The general methodologies used for morphological assessment could be divided into visual descriptions and metrical descriptions of the palatal configuration. To elucidate possible mediating and interactive effects on alterations of palatal development in preterm infants, the analysis of studies was ordered as follows.

• Visual descriptions of the palatal configuration of PT/LBW infants

- Incidence of high arched palates

- Incidence of grooving

- Palatal morphology in relation to intubation time

- Duration of intubation associated changes

- Palatal morphology in relation to birthweight

- Palatal morphology in relation to weight at the time of impression taking

- Palatal morphology and characteristics of the tube

- Feeding tubes

- Palatal morphology and tube position

- Palatal morphology and palatal plates

* Palatal morphology and feeding plates

* Palatal morphology and protective plates

- Palatal morphology and oral functions

- Palatal and alveolar cysts

- Influence of positioning on the orofacial development of PT infants

• Metric descriptions of the palatal configuration of PT/LBW infants

- Palates of non-intubated PT infants

- Influence of intubation on the palatal configuration of PT infants

* Length of palate

* Depth of palate

* Asymmetry of palatal depth

* Width of palate

* Asymmetry of palatal width

* Crossbite

* Palatal contour in relation to GA

* Grooving in relation to weight

* Grooving in relation to characteristics of the tube

* Palatal configuration in relation to duration of intubation

* Duration of intubation-associated changes

* Palatal morphology and palatal plates

* Influence of diet on the development of the palatal dimension

* Influence of feeding mode on the development of the palatal dimension

* Influence of cranial form on the palatal dimension

• Comparison of non-intubated PT/LBW and term infants' palatal measurements

## Results and Discussion

Seventy-eight articles published between 1940 and 2000 were included in the analysis of descriptions of the palatal morphology and palatal development in preterm and low birthweight infants. The majority of the studies were published between 1980 and 1990.

### Visual descriptions of the palatal configuration of PT/LBW infants

#### Incidence of high arched palates

Very high arched palate, but 'no palatal groove' was seen in 32% of 37 VLBW infants aged 9 to 75 months (72% out of the 37 infants had been intubated for on average 34.5 days) [1].

#### Incidence of grooving (Table 1, see Additional file 1)

Alveolar grooving [2], and 'palatal grooving' [2-13] have been described as a complication in connection with oral intubation. They never occurred in combination [2]. The various hypotheses on the cause of grooving are discussed in Part 3. The majority of articles dealing with the phenomenon fail to give a definition of palatal grooving. However, there are three exceptions:

1. Two authors defined a palatal groove as follows: '*Narrow channel of variable depth located near the midline of the palate as identified by visual inspection of the maxillary cast*' [6] (Comment: Consider the variability of the term 'narrow').

2. Two other authors, performing intraoral measurement with a micrometer *'from its floor to the surface of the palate at the midpoint of the hard palate*', selected a palatal groove of ≥0.5 cm arbitrarily as significant [14] (Comment: Consider how difficult it is to make precise intraoral measurements in a tiny infant).

3. A further group stated: '*By definition, a palatal groove is an architechtural deformity of the palate caused by external pressure from the orotracheal tube*' [15].

Intubation does not invariably lead to grooving [16,17]. The incidence of palatal grooving in PT infants is quoted at 7 – 90% [6,14,16-18] (n. b. 'grooving' may also be a matter of thickened palatine ridges). Only a few cases of alveolar grooving have been reported [4,19,20]. The deepest of these alveolar grooves divided entirely the alveolar ridge [19]. No evidence concerning the resolution of the defects has been given.

#### Palatal morphology in relation to intubation time (Table 1, see Additional file 1)

Grooving may occur just 12 hours after intubation; the longer the intubation time, the greater the incidence of groove formation, with a percentage of 87.5% grooves with an intubation time of more than 15 days [6]. In six infants intubated 50 ≥ 89 days, no palatal deformity was detected [5]. Two case reports, however report large or deep palatal grooves after 70 – 95 days of intubation [12].

A significant correlation between severty of groove and length of intubation in a group of infants without protective plates was observed [7,8]. In one of the studies, however, a statistically significant difference among the examaniers was revealed [7], in the other the intra- and inter-examiner reliabilty is not given [8]. In contrast, Raval et al. [1] quoted that palatal arch morphology was not influenced by duration of ventilation (reliability of the method not given).

#### Duration of intubation associated changes

' ... *It is unknown whether the palatal groove is permanent *... ' [6] (Table 1, see Additional file 1). While an almost complete resolution of a palatal groove after an unstated amount of time was reported in a letter [3], other authors did not find a noticable closure of a '*cleft*' at four months of age [5] (Table 1, see Additional file 1).

Watterberg and Munsick-Bruno [16,17] observed grooves in 90% of PT infants at the time of extubation. 67% [16], respectively 70% [17] still displayed the grooving six months after extubation, while the other had high arched palates (mean intubation time 67.6 days). The study had a high drop-off rate (Tables 1 and 2, see Additional files 1 and 2).

In the experience of some authors, the prominence of the lateral palatine ridges recedes after extubation, with a normal tongue motion ensuing and the palate having a normal appearance by the age of 2 years [2,21,22].

In an infant that had previously been intubated for 4 months a normal palatal contour in the second year of life was observed, whereas the groove was found to have increased in size in another patient at 21 months [13] (Table 2, see Additional file 2).

By the age of 2 – 5 years, no grooves were observed in 31 previously intubated PT infants in the clinical section of a follow-up study [23] (Table 2, see Additional file 2).

In contrast, in children of the same age group palatal grooving, a high palatal vault and crossbite were found in 28%, 100% and 16%, respectively in another study [24]. The persistence of a palatal groove acquired in the neonatal period for as long as 5 years was observed [25] (Tables 1 and 2, see Additional files 1 and 2).

In 32% of 37 VLBW infants aged 9 – 75 months very high arched palates have been diagnosed (72% of the 37 infants had been intubated for an average of 34.5 days) [1] (Table 1, see Additional file 1).

In 27 four year old VLBW children an equal amount of crossbite (15%) compared to normal controls was found [26].

#### Palatal morphology in relation to birthweight

Comparing three studies [1,23,24] with inclusion of children with increasing birthweights the neonates in the study with the lowest mean birthweight had the highest incidence of palatal deformation, i.e. 37% very high arched palates [1], while the probands in the study with the highest birthweights had no palatal deformity (Table 3, see Additional file 3).

#### Palatal morphology in relation to weight at the time of impression taking

No correlation between weight at the time of impression-taking and the incidence of grooving has been reported [6]. No data was given on age, GA and BW of the infants (Table 1, see Additional file 1).

#### Palatal morphology and characteristics of the tube

In conclusion to two case reports on deep palatal grooves, Saunders et al. [12] stated: '*Although chemical contents of previously used endotracheal tubes have been shown to cause some tissue irritation, the tubes used were made of polyvinyl chloride *... *and are unlikely to be the major cause of this injury*' (i.e. the grooves) (Table 1, see Additional file 1).

In contrast, other authors argue: '*The rigidity of these PVC tubes is believed to be strongly related to the development of the grooves in the intubated neonates*' [27] (Table 1, see Additional file 1).

Warwick-Brown [13] considered that '*the detrimental effect of the orotracheal tube versus orogastric tube may be a reflection of its increased size and rigidity and/or its use in the very early postnatal period*' (Table 2, see Additional file 2).

#### Feeding tubes

Infants of 26 to 32 GW have neither a sucking nor a coordinated swallowing reflex, thus the normal pattern of feeding is impossible because of the risk of inhalation and asphyxia [28]. A nasogastric feeding tube is commonly used to feed PT infants, until the sucking reflex is observed at about 36 weeks gestation age, and some infants require tube feeding even after this point [29].

The neonatal infant is an obligatory nose-breather. In normal circumstances this is a favourable characteristic as it allows the child to suckle while breathing through the nose [28]. Nasogastric feeding tubes, however obstruct the nares; this increases both the danger of hypoxia and the work of breathing [29]. Orogastric tubes used for a period of up to 50 days induced no grooves in 94.3% of cases [6] (Table 1, see Additional file 1). Arens and Reichman [30] described grooving in three VLBW infants at 85, 65 and 65 days after insertion of no. 5 F polyethylene feeding tubes for 108, 75 or 65 days. Simultaneous oral feeding was performed for 15, 26 and 9 days. The grooves were still present at 12 months in 2, at 13 months in 1 infant (Table 1, see Additional file 1).

#### Palatal morphology and tube position

It has been postulated that the effect of the tube is dependent on its position and the developmental state of the palate-forming bone and that most likely the palatal groove forms due to the continuous pressure of the endotracheal tube against the median palatine suture [6]. Some authors therefore recommended shifting the tubes to one side to prevent grooving [5]. However, one study revealed that grooving occurred even with laterally positioned tubes. The author attributed this to insufficient tongue thrust against the palatal shelves, allowing the shelves to grow together [22]. Even a laterally positioned tube may exert pressure in the rear palatal area, also giving rise to grooving [31].

#### Palatal morphology and palatal plates

##### Palatal morphology and feeding plates

According to two case reports an acrylic feeding plate, which is sometimes used to fix the orogastric tube, cannot protect the palate against the earlier assault of an orotracheal tube [13] (Table 2, see Additional file 2).

##### Palatal morphology and protective plates

Passing tubes through the mouth causes discomfort to child, shown by an increase in movements of the jaw and tongue [28]. In order to stabilize oral ventilation or feeding tubes against displacement from tongue and jaw movements, and thus against accidental extubation, to remedy palatal narrowing or grooving and protect primary teeth from trauma caused by intraoral tubes denture like protective plates are recommended or used by various authors [2,7,8,13,15,24,27-30,32-35]. Such an oral plate has been recommended for any infant requiring an oral tube for more than 24 hours, since 12 hours was the shortest period for palatal groove formation (no information concerning size, depth or severety of that groove was reported) [27].

A 90% reduction of spontaneous extubation and a 100% succes in prevention of palatal groove formation was reported in 30 intubated preterm infants with protective plates; babies who are receive this appliance should be medically stable as determined by the attending neonatologist [15].

In a randomized study a prefabricated palatal stabilizing device was compared with an acrylic, custom-made palatal stabilizing device. In those 34 PT intubated children with the prefabricated device, the appliance turned out to be significantly less retentive, thus requiring a greater monitoring. Accidental extubation occurred significantly more often than in 36 intubated preterm infants with a custom-made appliance. Both groups were medically stable and did not differ statistically significant with respect to birth weight, gestational age and period of intubation [33].

The plates are fabricated in the dental laboratory after an impression is taken from the infants mouth. The act of inserting and seating the tray with the impression material is often associated with a slight increase in the heart rate as displayed on the ECG monitor, however, once the tray has been seated in place, the vital signs remain within normal limits [15,28]. To preserve the child in its favorable environment, impressions are taken while the baby is in the incubator. This complicates the procedure but has the advantage that the infant's well being can be monitored throughout the impression procedure. To make the impression taking process more secure and to avoid the danger of indigestion or aspiration of the impression material, it was recommend to insert the impression taking material with the tray into a condom prior to insertion into the mouth [34]. A covered '*chimney*' in the acrylic plate corresponding to the size of the prospective tube may have the advantge of a sticking plaster to fix the tube, which can be the cause for dermal irritations to be unnecessary. On the other hand, this construction holds the plate in place without fixative cream being necessary [34]. Other authors retain the appliance in the mouth by means of adhesive powder [15].

Those plates, which should be secured by dental floss taped to the newborns cheek to prevent aspiration have proved capable of resisting a displacing force of 150 – 200 g, which is more than adequate to support two feeding tubes in position [28,29]. The device should be removed daily for cleaning [28,36] and can be readily manipulated by the neonatal nursing staff [27,28,36]. Care of the infant with such a device is not particularly different from care of babies without such a device [32]. Infants are reported to tolerate the procedure and the appliance well [28,29,35].

Some authors recommend to fabricate a new appliance after 2–3 weeks to allow for growth [29], others make a new plate approximately every 4 weeks [15,27]. Religning of the appliance at an interval between 10–20 days may extent its fit for up to 6–8 weeks [28,36].

In only one study, attention has been made to instances of ulceration or erythema to the palate [28]. Both could be denied. Neither was evidence found for monilial infection, probably because in these infants no food is taken by the mouth, so there is no possibility of food substances contaminating the palate [28]. A retrospective review of the infection rates showed no significant increase in the incidence of nosocomial infections during the period palatal stabilizing devices were used [15].

Preliminary investigations suggested that the appliance may have an important part to play in reducing the breathing effort necessary in 'at risk' premature infants and in lowering the arterial *CO*_2 _levels in hypercapnic children [28]. Regrettably, no studies concerning this subject were found during the literature searching process.

#### Palatal morphology and oral functions

Some orally intubated infants suck energetically at the tubes [5,37], thus shaping the oral tissues to the insertion direction of the tube in addition. This hypothesis is supported by the finding that in term infants a sucking habit narrows the palatal width significantly [38].

In PT infants an aberrant feeding pattern was observed, which might be the cause or else the consequence of a change in the palatal morphology of PT infants [39,40].

#### Palatal and alveolar cysts

The prevalence of palatal cysts is significantly lower in the PT infant (9%) (examined 12 days after birth) than in the term infant (30%) (examined 1 day after birth), as is the prevalence of maxillary anterior alveolar cysts (PT 27% vs. term 58%); palatal and maxillary alveolar cysts increase with increasing gestational age, post-natal age and birthweight; no significant differences were found in the prevalence of palatal and alveolar cysts for gender, nor while comparing caucasian preterm infants with a group of non-caucasian infants rising from black, latino and indian children [41].

#### Influence of positioning on the orofacial development of PT infants

It was stated that ' ... *Positioning and gravitational forces may interrupt or cause deviation in the development of palatal, cranial and facial bones*' [42]. '*The effect of these changes tends to alter the facial appearance of a child*' [43,44].

### Metric descriptions of the palatal configuration of PT/ LBW infants

Twelve metrical studies dealing exclusively with PT or LBW infants' palates were found [10,14,23,32,37,42,45-50] (Table 4, see Additional file 4). One had the exactness of different measuring methods as the primary interest of outcome [42]; three examined the effect of protective appliances [32,37,49], four included preschool or school children of a wide age range [10,23,45,48] (n. b. the mean difference in palatal width from 9 – 12 years in girls has been reported to be 0.9 *mm *in the molar region [51]); one measured palatal depths intraorally, entailing the risk of being imprecise [14]; a further study included term and PT infants [50]. In the majority of studies a problem with the reliabilty of the measuring method was present: Either the reliabilty was not given [14,46-48,50] or a significant measuring error for palatal depth was recorded [37], or the coefficent of variation for repeated palatal height measurements ran up to 11.73% [42,49].

Additionally to the above mentioned studies, the authors of the review recalculated data given in two doctoral thesis, and therefore were able to 'extract' figures concerning preterm infants from studies which primaraly included both, preterm and term infants [52,53] (Table 5, see Additional file 5; Part 1: Table 3, see Additional file 3 of Part 1). The measuring method and the reliability of the method was not given in either of the two latter studies. For the above mentioned reasons, criteria for a systematic analysis was not applicable to the retrieved publications.

#### Palates of non-intubated PT infants

Grooving with humping-up of the lateral palatal margins seen in infants following orotracheal intubation was not observed in any non-intubated infant [37] (Table 4, see Additional file 4).

Recalculation of the data given by Neumann [53], including exclusively spontaneously delivered children (occipito-anterior vertex presentation) revealed no significant difference in palatal width of preterm infants with respect to gender (Table 5, see Additional file 5; Part 1: Table 3, see Additional file 3 of Part 1). A comparison of palatal depth measurements at 36/37.6 and 53/53.8 weeks postmenstrual age reveals a lower palatal depth in non-intubated children [47] compared with intubated children [50] (Table 4, see Additional file 4). This difference between intubated and non-intubated is even more pronounced than expressed by the comparison of the pure figures, taking into account, that in the latter study palatal depth was measured from the lateral alveolar ridges, which are 'lower' than the alveoar crests, from where the measurements of the former study were conducted.

For 6 LBW children which were included in the study of Klemke [52] the authors of this review found a significant correlation between maximum palatal width and body length (Pearson, one sided, *p *= .009).

#### Influence of intubation on the palatal configuration of PT infants

##### Length of palate

Anterior palatal length (measured between the midpoint between the junctions of the lateral grooves with the gingival grooves and the anterior midline point on the alveolus) and maximum palatal length (distance between the midline point on the anterior part of the alveolus and posterior limit of the gingival grooves) were similar for non-intubated and intubated PT infants until term. The presence of prolonged intubation thus had little effect on the increase in length of the preterm palate [37]. The study ended at term.

##### Depth of palate

One study was excluded for that point, as a significant error of the method has been described by the authors for palatal depths measurements [37] (Tables 4 and 5, see Additional files 4 and 5).

The results of the literature research with respect to palatal depth were heterogeneous (Tables 6 and 7, see Additional files 66 and 7). Procter et al. [50] found only a small and transient effect of oral intubation on palatal depth, which disappeared at term (only 4 infants were intubated > 10 days, term infants were included in that study) (Tables 4 and 5, see Additional files 4 and 5). Visual inspection of the casts in that study revealed that palatal grooving did not always correspond with relative palate depth, but did usually occur in intubated infants. Procter et al. [50] therefore concluded that palatal grooving is not caused by the direct pressure of the orotracheal tube but is more likely to be due to overgrowth of the lateral palatine ridges. Whether the cause is irritation by the tube or the impairment of a normal tongue function could not be clarified within the framework of their study. At extubation time, the incidence and severity of grooves was found to be closely related to BW and total intubation time (mean intubation time > 15 days) [14] (Table 1, see Additional file 1).

Fadavi et al. [32] reported the deepest indentations of the palate in children, who had been intubated for more than 30 days; the prevalence of oral defects increased with increasing intubation time as with decreasing BW and significantly greater palatal depths were recorded in 2 – 5 year old, formerly orally intubated children (mean intubation time 36 days) [45] (Tables 4 and 6, see Additional files 4 and 6). Significantly higher palatal vaults and grooved palates in 3 – 5 and 7 – 10 years old formerly intubated PT children compared to non-intubated term children were also described by other authors (mean intubation time 18.3 and 26.4 days, respectively) [48] (Tables 2, 6, 7, see Additional files 2, 6, 7). Significantly higher palatal depths in the anterior region of formerly intubated children at a mean age of ten were measured (mean intubation time 15.2 days) [10] (Tables 4 and 7, see Additional files 4 and 7). Seow et al. [23] were the only group of authors who did not report any indentations of the palate in association with orotracheal intubation, the intubation time of the sample of that study was the shortest (Table 6, see Additional file 6).

##### Asymmetry of palatal depth

The only study on that point was excluded for that subject due to contradictory statements in text and tables [48] (Table 2, see Additional file 2).

##### Width of palate

Palatal width of intubated PT infants was reported to be significantly smaller in comparison to non-intubated PT infants from 32 weeks to term at the lateral grooves (mean intubation time > 30 days, study ended at term) [37] (Tables 2, 4, 5, see Additional files 2, 4, 5), and also in comparison to non-intubated term infants; the latter was true for the deciduous (Table 8, see Additional file 8) as well as for the mixed dentition (Table 9, see Additional file 9): Kopra and Davis [48] reported, that at ages 3 – 5 and 7 – 10 years the palates of intubated PT children were significantly smaller compared to age matched controls (mean orotracheal intubation times 18.3 and 26.4 days, respectively; mean orogastric intubation times 55.6 and 52.4 days, respectively) (Tables 2, 8, 9, see Additional files 2, 8, 9). This is confirmed by another study, in which the palates of formely intubated children at a mean age of ten years were significantly narrower, but only in the region of the second deciduous molars and first permanent molars (mean intubation time 15.2 days) [10] (Tables 4 and 9, see Additional files 4 and 9). Only a small and transient rise of palatal index (depth/width ratio), disappearing at term in children intubated for ≥ 10 days was described in another paper, (only 4 infants were intubated > 10 days, term infants were included in that study) [50] (Tables 4 and 5, see Additional files 4 and 5).

##### Asymmetry of palatal width

From the deciduous front teeth up to the first primary molar no palatal asymmetry was proven in 2 – 5 year old formerly orally intubated LBW children (mean intubation time not given) [23]. For the mentioned frontal region, this has been proven for 8 – 11 year old infants [10] (Table 4, see Additional file 4). In the region of the second deciduous and first permanent molars two other studies showed significantly more asymmetric palates of formerly orally intubated children compared to non intubated, normal controls with respect to palatal width proportional asymmetry for 3 – 5 year olds [48] and for 8 – 11 year olds [10] (Tables 2 and 4, see Additional files 2 and 4). In contrast to the latter authors, the former could not find significant differences in palatal width proportional asymmetry between 7 – 10 year old intubated and non-intubated children.

##### Crossbite

Tables 8 and 9 (see Additional files 8 and 9) show the crossbite frequency in the deciduous and mixed dentitions, respectively. Literature research revealed, that the crossbite frequency of intubated PT children did not differ significantly in all studies from that of term, non intubated controls: One study failed to show a significant difference with respect to crossbite in 2 – 5 year old, formerly orally intubated VLBW and LBW children compared to NBW controls (mean intubation time 36 days) [45] (Table 4, see Additional file 4). In contrast, significantly more crossbites in 3 – 5 year and 7 – 10 year old formerly orally intubated children were diagnosed compared to non-intubated controls by another research group (mean intubation time 24.6 days) [48] (Tables 2, 8, 9, see Additional files 2, 8, 9).

##### Palatal contour in relation to GA

With the same postmenstrual age up to < 40 weeks, relative palate depth tended to be higher in less mature children, but those were in fact the children with the highest percentage and duration of intubation. Depth and width of the palate were related to gestation and postmentrual age, with the most mature babies having the largest palates, but gestation had no effect on palatal index, i.e. the depth/width ratio [50] (Tables 4 and 5, see Additional files 4 and 5). Bias could have come over this study ≥ 40 weeks of gestation, as term infants had been included. Analysis of variance revealed significant relationships between high vaulted palate, palatal grooving, and gestation [48] (Table 2, see Additional file 1).

##### Grooving in relation to weight

Three studies claim that the development of grooving is closely tied to BW [14,45,48] (Tables 1, 2, 4, 5, see Additional files 1, 2, 4, 5). N. b. the confounding that probably the most immature infants need the longest intubation.

##### Grooving in relation to characteristics of the tube

The incidence and severity of grooving are not reported to be related to the consistence of the tube; even the use of soft tubes can thus not reduce the incidence and extent of palatal grooving [14]. Although the authors detected no difference in the incidence of commonly recognized complications of endotracheal intubation when hard and soft endotracheal tubes were compared, the flexibility of the soft tubes occasionally entailed a prolonged intubation time (Table 1, see Additional file 1).

##### Palatal configuration in relation to duration of intubation

Molding of the gum pad can be seen within hours of intubation [37] (Tables 2 and 4, see Additional files 2 and 4). The incidence and severity of grooving have been reported to be closely related to the total intubation time [14]. The authors invariably observed grooving in children below 1000 *g *bodyweight after 7 days intubation. No neonate developed a palatal groove when mechanical ventilation was continued for seven days or less (Table 1, see Additional file 1). In a letter referring to that paper, other authors [54] express their surprise, that the number of infants in whom palatal grooves developed who were intubated less than seven days was so low. The reply was that the low incidence was most likely tied to the older mean gestational age of the group ventilated for less than seven days [55].

A high palatal vault was recorded in 69% and palatal grooving in 25% of 2 to 5 year-old children, both parameters increasing with longer duration of intubation [45] (Table 4, see Additional file 4). Others found palatal height not to be affected by length of intubation [10,49], nor palatal width and area [49] (Table 4, see Additional file 4).

According to Procter et al. [50] prolonged intubation > 10 days only leads to a small and transient increase in relative palatal height (i.e. palatal index, i.e. depth/ width ratio) until term (Table 4, see Additional file 4). Bias could have come over this study, as only max. n = 4 infants had been intubated ≥ 10 days, and term and NBW infants had been included.

At the age of 2 – 5 years, no influence of duration of intubation on palatal symmetry could be proven from the frontal region up to the region of the first deciduous molars [56] (Tables 2 and 3, see Additional files 2 and 3). Accordingly, no differences between children aged 8 – 11 which had been intubated as neonates for ≤ 15 days and children intubated > 15 days with respect to palatal width asymmetry were recorded [10] (Table 4, see Additional file 4).

##### Duration of intubation-associated changes

Once the tube was removed, the gum pad remolded in most instances in one study [37] (Tables 2 and 4, see Additional files 2 and 4). At term no more influence of intubation on palatal depth was recorded in another study [50] (Table 4, see Additional file 4). Bias could have come over this study, as only max. n = 4 infants had been intubated ≥ 10 days, and term and NBW infants had been included.

In children aged 2 – 5 years in contrast, significantly greater palatal depths were revealed in orally intubated PT infants compared to age-matched NBW children [45]. Twenty fife percent of the children still had severe to moderate grooves (2/52 < 3 *mm*, 6/52 = 3 – 5 *mm*, 5/52 > 5 *mm*), and 69% very deep to deep palatal vaults. Only 25% had normal, and 6% flat or shallow palates [45] (Table 4, see Additional file 4). In contrast, in the same age group, no palatal grooves and no palatal asymmetry were detected in an intubated group compared with a non-intubated group with repect to reference points at the gingival margins from the central up to the first primary molars [23] (Table 4, see Additional file 4). No data was given as to how many of the previously intubated infants had grooves originally. The authors hypothesized: '*Growth changes and remodeling of the palate and alveolus in the first few years of life probably correct any deformation caused by laryngoscopy and endotracheal intubation. That growth changes can allow for remodeling of the palate is seen in patients' thumb or finger sucking habits. Most cases of uncomplicated palatal deformities resulting from digit sucking are resolved once the habit is discontinued*' (Table 4, see Additional file 4).

In 3 – 5 year old, formerly intubated PT children the following significant differences were found compared to age matched, normal controls: smaller palatal widths as well as a higher incidence of high vaulted palates and of palatal width proportional asymmetry in the molar region. No differences were described, however with respect to palatal depth and palatal width asymmetry [48] (Table 2, see Additional file 2). In 4 year old very low birthweight children an equal number (15%) of crossbites compared to NBW controls was diagnosed [26]. In 7 – 10 year old, formerly intubated preterm children, a significantly greater prevalence of a high palatal vault, grooved palate and crossbites was described, as significantly smaller palatal widths. Again, palatal depth did not differ significantly among the groups, neither did palatal width, nor palatal width proportional asymmetry [48] (Table 2, see Additional file 2).

In 8 – 11 year old children no significant differences in mean dental arch widths in the canine and molar region were measured while comparing formely intubated PT children and non intubated controls matched for age and gender [10] (Table 4, see Additional file 4). Like Seow et al. [23] (Tables 2 and 3, see Additional files 2 and 3), the former authors did not detect any significant differences in palatal asymmetry from the centrals to the first primary deciduous molars between intubated and non-intubated children. However, in gingival reference points located more distally (second deciduous molars and first permanent molars) they found significantly smaller palatal widths in formerly intubated vs. non intubated children (n. b. dental arch widths are measured at dental reference points, where buccal tipping of the tooth crown could compensate for a small transvere maxillary dimension, this buccal tipping could also compensate crossbites; palatal widths are measured at gingival reference points.). The left side of the palate in those intubated children was significantly wider than the right side posteriorly at the level of the second primary molars and first permanent molars [10] (Table 4, see Additional file 4). This could happen, because true stabilization of the endotracheal tube is only achieved extra-orally [57] and leaves open the possibilty for intra-oral tube displacement posteriorly towards the side of the prone nursing position, which is often on the right to aid gastric emptying. Prone sleep position has been shown to promote dolichocephyly and cranial moulding on the side on which the infant is nursed [58]. Macey-Dare et al. [10] also found the palatal heights anteriorly to be significantly steeper, but only at the level of the incisor region. Posterior crossbite or crossbite tendency was detected in only 11% of the formerly intubated children, what is comparable to that reported for the general population (7 – 10%) [59].

##### Palatal morphology and palatal plates

Anterior palatal length (midpoint between the junctions of the lateral grooves with the gingival grooves and the anterior midline point on the alveolus) and maximum palatal length (distance between the midline point on the anterior part of the alveolus and posterior limit of the gingival grooves) were similar for non-intubated and intubated infants as well as for intubated infants with protective plates [37]. A significant difference between a no-plate and a plate group in the percentage changes in lateral growth was recorded only for the anterior part of the palate at the lateral grooves, but not for the posterior region. There was a trend of palates of intubated children to be deeper, which was, however not significant. A significant error in the method of palatal depth determination was recorded in the study, which ended at term [37] (Table 4, see Additional file 4).

Palatal plates are able to protect intubated children from grooving [32] (Table 1, see Additional file 1). The effect of protective plates may consist in stabilization of the palate against deforming forces by the mattress rather than in keeping intraoral tubes in place [49]. However, only a poor correlation between cranial index and palatal index (depth/width ratio) was found [50].

Some authors see in the use of plates only a minimally protective effect: in their study population the mean difference in palate depth at 32 weeks between the intubated and non-intubated infants was 0.35 *mm *(only 4 infants had been intubated for ≥ 10 days) [50]. In comparison, the mean difference in palate depths at 32 weeks between protected and unprotected palates in the study by Ash and Moss [37] was 0.21 mm acording to Procter et al. [50], a statement which is incorrect: The mean difference in palate depth at 32 weeks between protected and unprotected palates in the study by Ash and Moss [37] at 32 weeks was 0.47 *mm*; furthermore, mean intubation time was longer in the latter study.

##### Influence of diet on the development of the palatal dimension

It has been reported that PT infants receiving human milk have lower bone mineralization rates than commercial formula fed infants [60]. This may lead to the hypothesis that commercial formula fed infants have an advantage in craniofacial and palatal bone growth and in resistance to postural deformation. However, any significant differences between breast- and formula fed children with respect to palatal width and depth [46] have been refuted (Table 4, see Additional file 4).

##### Influence of feeding mode on the development of the palatal dimension

Major benefits for growth in palatal width and palatal area followed the introduction of oral feeding [49]. The authors see the cause in the forming effect of the tongue on the shape of the palate during oral feeding. However, they also observed in PT infants an aberrant feeding pattern which they associated with palatal deformations.

##### Influence of cranial form on the palatal dimension

PT and LBW infants often have an unusually long, narrow head (dolicocephaly) compared with full term babies [43,61-63]. To prevent cranial deformation and thus any associated narrowing of the palate, the use of foam pressure dispersing pads (PDP) positioned on either side of the infant's head was recommended [49]. In comparison with a control group without pdp, a significantly greater change in the temporomandibular diameter was observed in the PDP group only in the early postnatal period prior to the start of oral feeding, but a significantly larger increase in palate surface and width only after the start of oral feeding. No significant intergroup differences were registered in the change in palatal height.

Although the cranial index (occipitofrontal : biparietal diameter; the greater the cranial index, the flatter the head) showed that the heads of the most immature infants were flattest and thus displayed side to side flattening, no significant correlation was detected between palatal index and cranial index [50]. Thus, the authors conclude that external pressure on the side of the head which causes head flattening cannot contribute to palatal grooving. Lateral head x-rays in children born small for gestational age showed a short anterior cranial base and small maxilla in a retrognathic face [64].

## List of abbreviations

[PT] preterm infant, [BW] birthweight, [LBW] low birthweight, [NBW] normal birthweight, [VLBW] very low birthweight, [NBW] normal birthweight, [GA] gestational age, [GW] gestational weeks, [NS] not significant

## Competing interests

The author(s) declare that they have no competing interests.

## Authors' contributions

AH designed the study, searched the databases, extracted the data, analyzed the results and wrote the manuscript. HR helped with study design, analysis and provided critical input in neonatal associated issues and revised the manuscript. UE and EH formulated the research question, helped with study design, analysis and in revising the manuscript. All authors read and approved the final manuscript.

## Supplementary Material

Additional File 1 Table 1 Incidence or severity of palatal grooving, relation to intubation time. Click here for file

Additional File 2 Table 2 Intubation-associated changes in palatal configuration and length. Click here for file

Additional File 3 Table 3 Influence of birthweight on palatal morphology of preterm / low birthweight infants. Click here for file

Additional File 4 Table 4 Measurements on palatal dimension of preterm infants. Click here for file

Additional File 5 Table 5 Crosstables for palatal measurements: preterm (LBW) vs. term (NBW). Click here for file

Additional File 6 Table 6 Metrical studies with respect to vertical palatal dimensions of intubated PT infants (deciduous dentition). Click here for file

Additional File 7 Table 7 Metrical studies with respect to vertical palatal dimensions of intubated PT infants (mixed dentition). Click here for file

Additional File 8 Table 8 Metrical studies with respect to transverse palatal dimensions of intubated PT infants (deciduous dentition). Click here for file

Additional File 9 Table 9 Metrical studies with respect to transverse palatal dimensions of intubated PT infants (mixed dentition). Click here for file
