# Supplementary material for: Palatal development of preterm and low birthweight infants compared to term infants – What do we know? Part 2: The palate of the preterm/low birthweight infant
Source: Head Face Med. 2005 Oct 28;1:9. doi: 10.1186/1746-160X-1-9 (PMC1298321; doi:10.1186/1746-160X-1-9)
Supplement: Additional File 1 — Table 1 Incidence or severity of palatal grooving, relation to intubation time. [file 1746-160X-1-9-S1.pdf]

Table 1. Incidence or severity of palatal grooving, relation to intubation time. Exclusion criteria was only given by [7].

studies	Study group / Control group / study design	Method and validity	Results
[24]	- 25 children, aged 2-5 Y, GA 29.3 +/-4.1 (range 24-32 W), mean BW 1089 +/- 420 g. (range 530-1828 g.), mean length of orotracheal intubation 34 +/- 28.2 D, range 7-90 D	- Dental casts, using a thermoplastic material.	- At 2- 5 Y, 100 % had a high palatal vault.<br>- 28 % had palatal grooving.<br>- 16 % had a posterior crossbite.<br>- Intubation ≤ 15 D: no grooving, no posterior crossbite.<br>- Intubation ≥ 30 D: higher incidence of grooving and posterior crossbite than those intubated ≤ 30 D
[30]	- 3 vlb. infants:<br>– 1: 870 g. BW, 26 W GA, 45 D of nasotracheal intubation, at 93 D attempts of oral feeding became successful<br>– 2: 1040 g. BW, 28 W GA, nasotracheal tube for 6 H, at 7 W of age partial oral feeding, at 75 D full oral feeding<br>– 3: 94 g. BW, 28 W GA, nasotracheal intubation for 15 D, supplemental oxygen by hood until 40 D, oral feeding initited at 56 D of age, withdrawal of orogastric tube at 65 D<br>– orogastric feeding tubes (no. 5, polyethylene) for 108, 75 and 65 D	- Visual inspection.	- Palatal groove after 85, 65, 65 D
[5]	- n = 1 PT infant (sex not given, 1049 g, GA 26 W)<br>- Orotracheal intubation at 1 minute of life, PDA at two D of age, ligated on 4 th D, orotracheal tubes were changed every 3- 7 D, patient died at 95 D of age.	- Probably visual clinical inspection.	- 'Deep palatal groove' at extubation .
[5]	- 1 M infant, 33 W GA, BW 1920 g.<br>- Intubation for 90 D (70 D with a tube of a diameter of 4.0 cm, four D 3.5 cm, 16 D 3.0 cm) and 6 D with nasotracheal tube, infant sucking on the tube on many occasions.	- Probably visual clinical inspection.	- High arched palate at 70 D, cleft at 90 D<br>- 16 weeks postextubation palatal deformity growing smaller.
[5]	- 1 F infant, 32 W GA, BW 990 g.<br>- Intubation with 3.0 mm Portex tube for 50 D	- Probably visual clinical inspection.	- At extubation (50 D) cleft of hard palate.<br>- At four MO no noticable closure of cleft.
[5]	- Six infants intubated > 50 < 90 D	- Probably visual clinical inspection.	- No palatal deformity.
[6]	- 63 neonates and infants who required orotracheal intubation for 1- 62 D prior to the making of the maxillary cast.<br>- Weight 580 g - 4400 g (42 <1500 g).<br>- 43 (68 %) had the maxillary impression taken during the first W of life.<br>- Sex, GA and BW not given.	- Impression of maxillary arch (thermal plastic compound, specially developed acrylic tray) (43 (68%) had the maxillary impression taken during the first W of life) .<br>- Master cast (dental stone).<br>- Visual inspection of palatal grooving (defined as a narrow channel of variable depth located near the midline of the palate).<br>- No validity of the method was given.	- Palatal grooving in 30 (47.6 %) infants;<br>- Intubation < 7 D (n=43) ⇒ 17 (39.5 %) palatal groove.<br>- Intubation 8-14 D (n=12) ⇒ 6 (50.0 %) palatal groove.<br>- Intubation >15 D (n= 8) ⇒ 7 (87.5 %) palatal groove.
[6]	- 106 neonates and infants (720-4500g.).<br>- (69 < 1500g) (48 (53 % < 7 D) with an orogastric tube for 1– 50 D.<br>- Sex, GA and BW not given.	- See above.	- Out of 106:<br>- 100 had no palatal groove at the time of the initial impression;<br>- Other 6 had previously had an orotracheal tube for up to 757 H
[32]	- 26 clinically stable PT, GA 24- 36 W, < 24 H of age, randomized in<br>– control group (n=14) without palatal stabilizing device (PSD) within 24 H of intubation, mean BW 941.4 +/- 271.8 g, mean duration of intubation 38 +/- 28 D and	- Postextubation palatal impressions on acrylic trays within 24 hours after removal of endotracheal tube.<br>- Observation and measurement of palatal	- No significant intergroup differences with respect to BW, mean duration of intubation (no data on comparison of groups with respect to GA given).<br>- None of children in PSD group showed any evidence of palatal

	– experimental group (n=12), with cold-cure methyl-methacrylat PSD, mean BW 989.2 +/- 379.2 g, mean duration of intubation 33 +/- 20 D	groove depth by using a boley gauge by a blinded dentist.<br>- Groove ≥ 0.5 cm was considered significant<br>- T-test for comparison of mean BW and duration of intubation.<br>- Correlation coefficient to analyze relationship between period of intubation and groove depth.	grooving.<br>- All 14 infants in control group experienced grooving from 2- 7 mm in depth: 64 % > 5 mm, 36 =% < 5 mm.<br>- Infants intubated > 30 D = most severe grooving patterns.<br>- Infants who were most at risk for palatal grooves = infants intubated > 2 W.<br>- Correlation coefficient between intubation period and groove depth $r = .92$.
**[7]**	- 60 VLBW infants without palatal abnormalties requiring orotracheal intubation (20 therefrom excluded (8 intubated < 5 D, 7 died, 5 required different endotreacheal tube)), thus randomized<br>- 22 children without palate plate (mean BW 852 g. (175), mean GA 26.7 W (1.7), average of 29.1 (21.1) D of intubation.<br>- 18 children with palate plate (mean BW 920 g (196), mean GA 27.7 W (1.6), average of 27.1 (18.7) D of intubation.	- Maxillary casts within 5 D of initial orotracheal intubation and within 1 W after extubation.<br>- Evaluation of casts by 3 blinded examiners.<br>- Grading of groove and averaging of results ((1=normal, 2=mild, 3= moderate, 4 = deep (>= 5 mm).<br>- Differences between groups (BW/ GA/ length of intubation): student t test.<br>- Differences between groups (impressions/ length of intubation): analysis of variance (two way classification, single observation).<br>- Length of intubation vs degree of groove Pearson correlation coefficent.	- No statistical significant differences between groups for mean BW, GA or length of intubation.<br>- Significant difference in rating for initial and final measurements between 3 examiners ($p < 0.05$).<br>- Difference (final-initial) did not differ significantly.<br>- No significant differences in the initial ratings of study and control group.<br>–Palatal grooves and differences between initial and final grading significantly smaller in the plate group.<br>–No correlation between severity of groove and length of intubation in the plate group.<br>–Significant correlation between severity of groove and length of intubation in the control group ($p < 0.05$).
**[8]**	- 40 VLBW neonates with orotracheal tubes randomized to<br>– control group (n=22), GA 27.1 +/- 1.6 W, BW 920 +/- 196 g., length of intubation 27.1 +/-18 D and<br>– study group (n=18), GA 26.7 +/- 1.7 W, BW 852 +/- 175 g., length of intubation 29.1 +/-21.2 D fitted with palatal appliance to protect maxillary arch from the orotracheal tube	- Palatal impressions within 5 D of initial intubation and following extubation.<br>- 3 blinded examiners, averaging of their results.<br>- Grading of grooves see above.<br>- Differences between groups (initial and final impressions and length of intubation): analysis of variance.	- No differences between groups for initial grading.<br>- Significant differences between final and final-initial gradings between groups ($p < 0,01$).<br>- Only in the control group did the severity of groove formation correlate with the length of intubation ($p < 0.05$) .
**[14]**	- Neonates out of a 12 MO period who required intubation and mechanical ventilation, < 2500g; patients < 800 g BW treated with a 2.5 mm internal diameter group, patients > 800 g BW treated with 3.0 diameter; tubes fixed to upper lip and cheek with tape.<br>- Study group: 57 neonates (standard PVC endotracheal tube; 'hard' group (identical internal and external diameters to the soft tube);<br>- Control group: 46 neonates with a modified 'soft' PVC endotracheal tube (identical internal and external diameters to the hard tube).	- Prospective, randomized, blinded study, weekly visual inspection for palatal grooving; in case a groove was present it was measured by a small micrometer from its floor to the surface of the palate at midpoint of the hard palate;<br>- Palatal groove ≥ 0.5 cm was chosen to be significant.<br>- If at time of extubation a groove was present it was photographed for comparison during follow up.	- No grooving when mech. vent ≤ 7 D.<br>- Palatal grooves regularly after 7 D in infants < 1000 g, neither incidence nor severety of palatal groove formation influenced by the kind of tube.<br>- Development of a groove closely tied to BW: <1000 g 50% (11/22) vs 17 % (3/17) >1000 g and total intubation time.<br>- Of the original 106 infants 3 were excluded due to technical failure with intubation; 4 deaths in the soft tube vs. 1 death in the hard tube group.
**[1]**	- 37 infants, BW 1024 +/- 228 g., GA 29 +/- 2 W<br>- 50 % M, 83 % black.<br>- 72 % intubated for 34.5 +/-28.7 D	- Blinded dental exam at ages 9- 75 MO.	- 32 % had very high arched palates, no palatal grooves.
**[12]**	- n = 1 PT infant (sex not given) with normal palate at birth (GA 26 W, with	- Probably visual clinical inspection.	- 'Large palatal groove' after extubation after 70 D

	orotracheal tube (changed every 3 to seven D) at three minutes of life, ventilation for 70 D		
**[16] + [17]**	- 40 PT, GA 25-37 W (mean 30.0 +/- 2.6).<br>- Intubated for 14- 243 D (67.6 +/- 51.1).	- Evaluation for palatal grooving by means of wax impressions of the palate at the time of extubation and six MO later.	- At the time of extubation:<br>- 37 (90%) with grooves.<br>- 3 without grooves (GA 29, 31, 31 W, intubated 24, 14, 14 D).

BW = birthweight, D = day(s), F = female, GA = gestational age, GW = gestational weeks; H = hour(s); LBW = low birthweight, M = male, MO = month(s), NBW = normal birthweight, NS = not significant, PT = preterm, VLBW = very low birthweight, W = weeks, Y = year(s).
