# Supplementary material for: Palatal development of preterm and low birthweight infants compared to term infants – What do we know? Part 2: The palate of the preterm/low birthweight infant
Source: Head Face Med. 2005 Oct 28;1:9. doi: 10.1186/1746-160X-1-9 (PMC1298321; doi:10.1186/1746-160X-1-9)
Supplement: Additional File 2 — Table 2 Intubation-associated changes in palatal configuration and length. [file 1746-160X-1-9-S2.pdf]

Table 2. Intubation-associated changes in palatal configuration and length.

<table>
<tr><th>studies</th><th>Patients</th><th>Method and validity of method</th><th>Results</th></tr>
<tr><td>[24]</td><td>–25 2- 5 Y old children, intubated for longer periods as neonates.
– GA 29.3 W (SD 4.1, range 24- 32 W).
– Mean BW 1089 g. (SD 420, range 530- 1828).
– Mean length of orotracheal intubation 34 D (SD 28.2, range 7-90).</td><td>– Impression with thermoplastic material, dental cast.</td><td>- All children had high palatal vault.
- 28 % had palatal grooving.
- 16 % had posterior crossbite.
- Children intubated ≤ 15 D did not have palatal grooving or posterior crossbite.
- Children intubated ≥ 30 D had a higher incidence of effects than those intubated ≤ 30 D</td></tr>
<tr><td>[30]</td><td>–<u>See Tab. 1.</u></td><td>- See Tab. 1.</td><td>– Groove still present at 12, 13 and 12 MO.</td></tr>
<tr><td>[37]</td><td>- See Tab 5.</td><td></td><td>– Once the tube was removed, the anterior gum pad remoulded in most instances.</td></tr>
<tr><td>[8]</td><td>–<u>See Tab 2.</u></td><td>- See Tab. 2.</td><td>– See Tab. 2.</td></tr>
<tr><td>[48]</td><td>–<u>Study group: PT, LBW, intubated (59 % Caucasian, 54 % male):</u>
– <u>43 3-5 Y old intubated LBW</u>
– Mean BW 992.6 g (SD 196, range 595-1247).
– Mean GA 28.9 W (SD 2.7, range 25- 36).
– Mean orotracheal intubation time 18.3 D (SD 21, range 1-99).
– Mean orogastric intubation time 55.6 D (SD 31, range 6- 169).
– <u>47 7-10 Y old intubated LBW</u>
– Mean BW 1044.1 g (SD 288.2, range 537-1616).
– Mean GA 28.7 W (SD 2.6, range 22-33).
– Mean orotracheal intubation 26.4 D (SD 31.4, range 1-99).
– Mean orogastric intubation time 52.4 D (SD 27.4, range 0-99).
–<u>Control group: mainly NBW, non intubated (56 % Caucasian, 51 % M):</u>
– 40 3-5 Y old (with four exceptions not intubated and full term, mean BW 3465.0 g. (SD 425.4 (range 2781-4264)).
– 44 7-10 Y old, full term, mean BW 3485.9 g (SD 495.4, range 1814-4173).
–<u>Exclusion</u>: history of intubation, developmental difficulties, facial abnormalties, neural dysmorphologies, constant finger habits.
–<u>drop out study group:</u> 59 %.
–<u>drop out control group:</u> 56 %.</td><td>- Identification of LBW and neonatally intubated children by medical records.
- Intraoral inspection (high vaulted palate = high palate configuration relative to palate size) +
- Alginate imprresions on pediatric stock trays, poured into stone models (to validate clinical assessment of high vaulted palate and palatal grooving).
- Asymmetry measured with a 3-dimensional rectangular cartesian coordinate system with respect to a fixed plane, comparison of values from reference points on contralateral sides to indicate discrepancies of the arch form in width and depth (3-5 Y old: on the lingual margin of central and lateral incisors and second primary molars; 7-10 Y old: correspondingly on the gingiva of the central and lateral incisors, canines and 6-vear molars. Measurements by locating the widest aspects of the tooth and reflecting this point to the gingival margin. <u>Palatal width asymmetry</u>: Difference between measurements of the distance of contralateral measuring points to the median palatal raphe. <u>Palatal depth asymmetry</u>: locating the midpoint on a line between each reference point and the raphe and then measuring the vertical distance to the palate. Difference between contralateral sides to determine palatal depth asymmetry.
- Vidicom Qualifier 1210 (accuracy 0.001 mm).</td><td>
<table>
<tr><td>3-5 Y olds</td><td>intubated (n= 41)</td><td>non-intubated (n=41)</td><td>* $p < 0.05$</td></tr>
<tr><td>high vaulted palate</td><td>63.4 %</td><td>10.0 %</td><td>*</td></tr>
<tr><td>grooved palate</td><td>14.6 %</td><td>0.0 %</td><td>* 1)</td></tr>
<tr><td>crossbite</td><td>22.0 %</td><td>5.0 %</td><td>* 1)</td></tr>
<tr><td>palatal width second deciduous molar region</td><td>24.02 mm</td><td>25.33 mm</td><td>*</td></tr>
</table>
<table>
<tr><td>7-10 Y olds</td><td>intubated</td><td>non-intubated</td><td>* $p < 0.05$</td></tr>
<tr><td>high vaulted palate</td><td>61.7 %</td><td>9.0%</td><td>*</td></tr>
<tr><td>grooved palate</td><td>23.4%</td><td>0.0%</td><td>*</td></tr>
<tr><td>crossbite</td><td>21.3%</td><td>2.3%</td><td>*</td></tr>
<tr><td>palatal width molar region</td><td>27.98 mm</td><td>30.16 mm</td><td>*</td></tr>
</table>
- Defects were not influenced by length of intubation.
- * = chi square test
- 1 = results of this analysis are not reliable due to low expected cell frequencies.
- Analysis of variance revealed significant relationships between high vaulted palate, palatal grooving, crossbite and both, BW and GA</td></tr>
</table>

**[49]**	–See Tab. 3.	- See Tab. 3.	–Duration of orotracheal intubation did not influence the degree of palatal deformation.
**[23]**	- 49 PT (23 m, 26 f)<br>- Ø BW 1213 g (range 605 g– 1500 g)<br>- Ø age at impression 3 Y, 3 M (range 26 M to 5 Y, 5 MO);<br>- 18 with endotracheal intubation[1];<br>- 31 without endotracheal intubation and mechanical intubation[1]<br>- Exclusion: neurological abnormalties, facial dysmorphology, clefts, finger sucking one Y prior to investigation, further intubation after leaving hospital after birth.<br>-<br>- [1] The authors give contradictory information on intubation, on pp. 33 and 34 of the article, they mention 22 intubated children.	- Dental examination.<br>- Stone models from compound maxillary impression on plastic spoon.<br>- Measurements at the primary lateral incisors, canines and first molars, by drawing tangents to the mesiopalatal and distopalatal surfaces and projecting a line bisecting the angle of the intersection to the gingival margin , reference line was median palate raphe.<br>- Reference point of each tooth at the gingival margin to determine symmetry.<br>- Olivetti inspector machine to determine the 3 dimensional coordinates of the reference points.<br>- Measurements to the nearest 0.1mm.<br>- Accuracy of the measuring method: SD 0.1-0.4 of the 3 measurements taken of any one model; difference between models NS.<br>- Location of reference points on the gingival margins of deciduous lateral incisors, canines and primary molars x, y and z coordinates of reference points determined directly by the Olivetti inspector machine, allowing measurements to the nearest 0.1 mm.<br>- Symmetry of the 2 models taken from each patient showed no statistical significant differences (level of significance: $p \leq 0.1$).	- No palatal grooves.<br>- No statistically significant differences between groups.<br>- No palatal asymmetry 2-5 Y after intubation.<br>- Length of intubation does not affect palatal symmetry
**[13]**	- N = 1 (f), 1230 g, GA 28 W, caesarean section; orotracheal tube for 8 D; Complications: pneumothorax and neonatal jaundice; at 8 D orogastric tube and acrylic feeding plate;	- Impressions at 8 D, 4 and 21 MO	- Normal palatal shape at 8 D; palatal V shape and 'deep groove' at 4 MO.
**[13]**	- N= 1 (m), 1050 g, GA 28 W, vaginal delivery; orotracheal tube for 4 MO, subsequently orogastric tube and acrylic feeding plate for 3 W	- Impressions at 4, 5, 6, 7, 10, 25 MO<br>- No further description of method given.	- 5 mm deep groove.<br>- After use of a feeding plate reduction in width but not depth of the groove.<br>- Groove persisted until 10 MO of age the (no change in depth but a progressive increase in width).
**[17]**	- See above.	- See above.	- Six MO after extubation 23 / 40 patients with grooves at the time of extubation returned:<br>- 16 (70 %) had persisting grooves (mean GA 30.5, SD 2.8, intubated mean 86.3 D, SD 62.5).<br>- Other 7 (30%) (mean GA 30.3 W, SD 3.2, intubated 24- 154 D, mean 56.3+/-44.2) had high arched palates.
**[16]**	- All neonates in the NBICU intubated >2 W;<br>- 40 PT neonates.<br>- GA 25-37 W (mean 30.0 +/- 2.6).<br>- Intubation for 14-243 D (67.6 +/- 51.1).	- Wax impressions of the palate at the time of extubation and six MO later.	- Six MO after extubation 18 / 40 patients with grooves at the time of extubation returned:<br>- none of the palates appeared normal.<br>- 12 (67 %) had persisting grooves (mean GA 31.0 W, SD 2.7, intubated mean 81.6 D, SD 49.9);<br>- the other 6 (33%) (mean GA 30.7 W, SD 3.3, intubated 24-154 D, SD 57+/-48.4) had high arched palates.

BW = birthweight, D = day(s), F = female, GA = gestational age, GW = gestational weeks; H = hour(s); LBW = low birthweight, M = male, MO = month(s), NBW = normal birthweight, NS = not significant, PT = preterm, VLBW = very low birthweight, W = weeks, Y = year(s).
