# Supplementary material for: Palatal development of preterm and low birthweight infants compared to term infants – What do we know? Part 2: The palate of the preterm/low birthweight infant
Source: Head Face Med. 2005 Oct 28;1:9. doi: 10.1186/1746-160X-1-9 (PMC1298321; doi:10.1186/1746-160X-1-9)
Supplement: Additional File 3 — Table 3 Influence of birthweight on palatal morphology of preterm / low birthweight infants. [file 1746-160X-1-9-S3.pdf]

Tab. 3. Influence of birthweight on palatal morphology of preterm / low birthweight infants.

studies	[24]	[1]	[56]
**N**	– 25	– 37	– 49
**mean BW (g.)**	– 1089 +/- 420	– 1089 +/- 228	– 1213 (range 605-1500)
**GA at birth (weeks)**	– 29.3 +/- 4.1	– 29 +/-2	– not given
**% of children intubated**	– 100	– 72	– 36 or 44.9 or 63.3 or 53.1 (author gives contradictory information on pages 30, 32 and 34)
**D intubated**	– 34 +/-28.2	– 34.5 +/- 28.7	– 1-64
**Age at examination**	– 2-5 Y	– 9 M – 6.25 Y	– 2.2- 5.4 Y
**palatal deformation**	– 28 % grooving	– 37 % very high arched palate	– 0 % palatal grooves, insignificant differences between intubated and non- intubated children with respect to palatal asymmetry

(BW = birthweight, GA= gestational age, MO = month(s); D= day(s), Y= year(s); M = male, F = female).
