# Supplementary material for: Palatal development of preterm and low birthweight infants compared to term infants – What do we know? Part 2: The palate of the preterm/low birthweight infant
Source: Head Face Med. 2005 Oct 28;1:9. doi: 10.1186/1746-160X-1-9 (PMC1298321; doi:10.1186/1746-160X-1-9)
Supplement: Additional File 4 — Table 4 Measurements on palatal dimension of preterm infants. [file 1746-160X-1-9-S4.pdf]

Table 4. Measurements on palatal dimension of preterm infants.

studies	Study group / study design	Method and validity of method
**[37]**	-See Tab. 5.<br>-Prospective longitudinal study.<br>-n= 45 PT < 30 W GA;<br>-3 groups:<br>-15 babies requiring orotracheal intubation were after random selection fitted with palatal protective appliances as soon as possible after birth and throughout the intubation period (32.1 D (19.3)), (GA at birth 26.6 (1.75) W, BW 0.86 kg (0.176)), (plates replaced all 10-14 D to allow for growth)<br>-Other 15 intubated babies requiring orotracheal intubation 30.8 D (25.5 D) without protective plates (GA at birth 26.2 W (1.94), BW 0.95 kg (0.23)<br>-All intubated babies were similar in sex distribution, BW and GA at birth.<br>-Controls: 15 non intubated babies (GA at birth 29.7 (1.5), BW 1.42 kg (0.36))	- See Tab. 5.<br>- Impressions (polyether material with a predominance of catalyst to hasten the set on stock impression tray) of the palate "as soon after birth as possible", at 32 W GA and at term.<br>- 50/50 stone plaster mix casts; for the palate plate group a second model was cast to construct the protective appliance;<br>- Measurements with a reflex microscope coupled to a pc;<br>- Test of the accuracy of the impression technique by repeated impressions on the same occasion at 6 randomly chosen babies (paired t-test): differences between the impressions NS<br>- 25 % of the models were measured on a different occasion and tested with a paired t-test to indicate the error of the method.<br>- <u>For palatal depth measurements a significant error at the 1.4. % level was found!</u>
**[45]**	– See Tab. 5.<br>– Inclusion:<br>– <u>Study group:</u><br>– n=52 children of the neonatal unit of the University of Illinois Hospital (1985- 1990), mean BW 1151 g (SD 418.3), mean GA 29.4 W (SD 3.4) intubated at least 24 H (mean 26 D (24.5), aged 2- 5 Y, a majority with hyaline membrane disease and subsequent bronchiopulmonary dysplasia .<br>– <u>Controls:</u><br>– 45 NBW children matched for age (only for palatal depth).<br>– <u>Exclusion:</u><br>– History of craniofacial surgery, congenital abnormalties and syndromes or orthodontic treatment.	- See Tab. 5.<br>- Four investigators.<br>- Clinical examination +<br>- Plaster casts from alginate impressions.<br>- Assessment of palatal dimension similar to that used by Shellard et al. (1986) and advocated by Klami et al. (1979).<br>- Measurements by an adjustable template.<br>- Depth: measured from a line connecting left and right interproximal areas of the first and second primary molars, at the gingival margin, perpendicular to the palate by 4 investigators (interexaminer measurements did not differ significantly from the sample mean (α= 0.01 (t-test)).<br>- Palatal grooving: tracing the cross section of the plaster cast in a transverse direction (at the same location where palatal depth was measured); measurements with a flexible plastic ruler from the deepest point of the groove to an imaginary line crossing the palate; 2 examiners, in case of different ratings repetition of the evaluation until agreement was reached.<br>- Palatal shape and contour (palatal vault): cross sections of the palate, creating a matrix of very deep, deep, shallow or flat palate (in case of different ratings repetition of the evaluation until agreement was reached)<br>- Posterior dental crossbites: evaluation of plaster casts.<br>- Accuracy of the method not given!
**[15]**	- See Tab. 1.	- See Tab. 1.
**[46]**	- See Tab. 5<br>- Prospective, interdisciplinary, longitudinal study.<br>- 26 non orally intubated PT infants, GA at birth 30.8 W (2.76), BW 1468 g. (531), divided into a breastmilk fed and a commercial formula fed group matched for corrected age and weight.<br>- Impressions at 36.8 W (1.4) and 52.6 W (7.16) postmenstrual age.	- See Tab. 5.<br>- Impressions.<br>- 3 measurements with a 3 D laser digitizer.<br>- Evaluation of nutrition, calcium and phosphate substitution and weight by patients´s records and history.<br>- Accuracy of the method not given.
**[47]**	- See Tab. 5.<br>- Prospective, interdisciplinary, longitudinal study.<br>- 27 PT infants, GA at birth 30.7 W (2.8).<br>- Impressions at 37.6 W (3.15) (n=27), and at a corrected age of 13.8 W (5.9).	- See Tab. 5.<br>- Impressions.<br>- 3 measurements with a 3 D laser digitizer.<br>- Accuracy of the method not given.

**[52]**	-See Tab. 5 and Tab. (3 Part 1)	- See Tab. 5 and Tab. 3 (Part 1)
**[48]**	- See Tab. 2.	- See Tab. 2.
**[10]**	- Inclusion: Caucasians without craniofacial anomalies, without habit activity, intact dentition with deciduous canines to second primary molars present (remark of the authors: no information was given if the children did or did not have orthodontic treatment).<br>- 43 PT, LBW children (GA 20-37 W, BW 957- 2040 g) from Oxford.<br>- Mean age 10 Y (range 8.4-11.1)<br>- Mean intubation time 15 D (range 1- 58) (23 <=15 D, 20 > 15 D)<br>- All fed orogastrically<br>+<br>- 50 NBW, term children (GA 39-41 W, BW 2650- 3970 g., aged 8.9-10.8 Y) from Oxford, individually matched with respect to age and gender, without history of intubation or orogastric feeding.	- Alginate impressions, wax bite registrations, cast within 24 H with a 50-50 % plaster and white stone. Remark of the authors: alginate loses its dimensional stability after a few H, thus incorrectnesses of the casts´ dimensions might have resulted.<br>- Width, length and height measurements recorded as values of the x, y and z coordinates of the cartesian scale.<br>- Marks for sagittal (median palatal raphe according to Lebret (1962) [66]) and horizontal reference planes (gingival alveolar margins of the deciduous and permanent teeth according to Kopra and Davis (1991) [48]) on the dental casts, from these the measurements were taken.<br>- Reference points: canine tips or <u>estimated</u> canine tips.<br>- Mesiobuccal cusp tips of first permanent molars.<br>- Tangents to the mesiopalatal and distopalatal tooth surfaces, then projecting a line bisecting their angle of intersection to the gingival margin.<br>- Grid markings of a clear plastic template ligned up with reference gingival reference points and raphe, mark (0.2 mm lead pencil (Morris et al. (1993) [42] at the perpendicular intersection of the two on the median palatal raphe.<br>- Width measurements: where perpendicular line from a reference point crossed median palatal raphe.<br>- Depth measurements: from horizontal reference plane to palatal raphe.<br>- Crossbite classification according to [65].<br>- Measurements with reflex microscope (3 dimensional digitizer with stereoscopic vision, x 20 magnification, light dot of 10 µm for landmark identification, measurements to the nearest 0.001 mm (remark of the authors: does this make sence while using a 0.2 mm pencil for marks on the model?).<br>- Error of method by double determination of the models and calculation by Dahlbergs formula = 0.111- 0.211 mm.<br>- No siginificant differences between genders, therefore males and females pooled.<br>- Independent t tests for differences in effects of length of intubation, palatal widths and heights, one way analysis of variance for arch widths, sample t-test for palatal asymmetry, no statistical tests for crossbites due to small numbers.
**[14]**	-See Tab. 1.	- See Tab. 1.
**[42]**	- Study conducted exclusively to compare the different measuring methods (see right column).	- 1. Stereophotogrammetry (binocular vision + analytic plotter).<br>- 2. Vernier callipers.<br>- 3. Olivetti inspector machine (allows direct measurement of dimensions on the three Cartesian axes on a millimeter scale with an SD of 0.1 – 0.4 mm and reading of measurements directly on a display panel [42].<br>- Stereophotogrammetry was found to be the most consistent method of measuring the palatal configuration of preterm infants. Vernier calipers significantly overestimated the height and underestimated the width, and were therefore considered not to be suitable for VLBW infants. Compared to stereophotogrammetry, the Olivetti inspector machine significantly underestimated the width [42].
**[49]**	- See Tab. 5.<br>- Inclusion:<br>- Total of 54 infants.<br>- <u>Intervention group:</u> n= 31, BW 1110 g (227), GA at birth 28.5 W (1.4), mechanically	- See Tab. 5.<br>- Temporomandibular diameter (TMD) (horizontal distance between the temporomandinular joints) taken with dividers, measured with a caliper calibrated to 0.1 mm (repeated measurements 2 h apart from 10 VLBW not included indicated repeatabilty r=0.95).

	ventilated for 240 H (362), fitted with a pair of pressure dispersing pads (pdp), of high density lightweight cushion designed to fit the lateral aspect of the head to relieve direct pressure from the zygomatic bones, asssociated structures and ear, while dispersing pressure across temporal, parietal and mandibular structures; continuously worn after the initial measurement, changed every 7 D.<br>- Controls: n= 23, BW 1215 g (215), GA age at birth 29.2 W (2.2), ventilated for 165 H (532) without pdp.<br>- No significant difference between the groups in BW, GA, duration of oral intubation, type of milk intake, in terms of subject age at each test measure and number of D between test measures.<br>- 2 phase study over 20 months.<br>- Test 1: 2- 7 D after birth, when moulding from delivery disappeared and infant´s condition was stable.<br>- Test 2: 1 D prior to commencement of oral feeding.<br>- Test 3: 1 or 2 D prior to discharge or at 40 W.<br>- Exlusion:<br>- 5 of 68 infants meeting the criteria for initial inclusion were excluded because of a grade 3 intraventricular haemorrhage (possibility of aberrant head growth + loss due to transfer to other centres).	- Compound palatal impressions by 1 single investigator (palatal impression was not taken if an orotracheal tube was in situ at a particular testing time).<br>- Stone cast poured from each palatal impression.<br>- Stereophotogrammetry to measure parameters of the palatal cast.<br>- From the computer file of coordinates, measurements of maximum width, maximum height and area were calculated.<br>- Assessment of bone metabolism was not performed but the type of milk intake.<br>- Statistics: version of ANOVA (general linear models procedure), which took in account any missing variables due to an orotracheal tube in situ, medical instability or earlier transfer to regional hospitals, and tested the influence of oral intubation on outcome<br>- Student`s t test showed statistically significant differences for birth parameters, initial craniofacial and palatal measurements and total H of orotracheal intubation.<br>- Contingency table was used to explore differences in nutritional intake.<br>- P < .05 level of significance.<br>- Study examined changes within time:<br>- Period 1: from test 1 to test 2.<br>- Period 2: from test 2 to test 3.<br>- Period 3: covered the whole study period.
**[53]**	-See Tab. 3 (Part 1), here: 20 PT infants, see Tab. 5.	- See Tab. 3 (Part 1), here: 20 PT infants, see Tab. 5.
**[50]**	- See Tab. 5.<br>- Prospective, longitudinal study.<br>- 76 neonates, GA 25-41! W (median 33)<br>- BW 715-4730 g! (median 1880 g)<br>- 23 infants ventilated 1- 32 D (endotracheal tube; Portex Blue);<br>- 19 intubated < 10 D<br>- 4 intubated ≥ 10 D<br>- Infants with need of intensive care nursed prone, others mostly supine.<br>- Nasogastric tubes used for delivering lavage feeds.<br>- Division into 3 groups:<br>- 27: < 32 W of GA (21 infants ventilated);<br>- 29: 32- 35 W of GA (2 infants intubated (< 10 D);<br>- 20 > 36 W of GA (none intubated);<br>- Controls: 15 PT non intubated babies (it was not possible to obtain a strictly matched control group, thus the mean BW and state of GA was higher for the control group; time interval between initial impressions and those at 32 W GA was shorter in the control than in the intubated groups).	- See Tab. 5<br>- One investigator.<br>- Vinylpolisiloxone impression (Elite fast).<br>- Stone hard plaster model (n=267).<br>- 267 impressions attempted; in 10 cases no impression was possible due to non compliance, in 40 the landmarks could not clearly be identified ⇒ 217 casts were included in the study.<br>- Palatal depth: at the deepest point of the palate (identified by visual inspection) relative to the alveolar crest.<br>- Palatal width: perpendicular to a line constructed by the midline point of the anterior crest of the alveolar gum pad ridge (identified by visual inspection) and the deepest point of the palate.<br>- Measurements at 28, 32, 36, 40, 53, 66, 92 W of postmenstrual age.<br>- Accurate identification of palatal width required that the model was held in a specially constructed spring loaded deck with a transparent upper deck that rested on the alveolar crestal margins, and had 0.5 mm holes drilled at 3 mm intervals along the sagittal and coronal planes; hole at the intersection lines was aligned perpendicular to the deepest point using a 0.5 mm pencil lead inserted through the hole vertically to the deepest point. By aligning the holes in the sagittal plane of the deck with the midpoint of the alveolar ridge the coronal plane on the palate model was marked and the points for measurement of palatal width identified.<br>- Measurements with a high precision reflex microscope (resolution of 0.2 m!).<br>Reliability of the method not given.
**[23]**	-See Tab. 2.	- See Tab. 2.

BW = birthweight, D = day(s), F = female, GA = gestational age, GW = gestational weeks; H = hour(s); LBW = low birthweight, M = male, MO = month(s), NBW = normal birthweight, NS = not significant, PT = preterm, VLBW = very low birthweight, W = weeks, Y = year(s).
