# Supplementary material for: Palatal development of preterm and low birthweight infants compared to term infants – What do we know? Part 2: The palate of the preterm/low birthweight infant
Source: Head Face Med. 2005 Oct 28;1:9. doi: 10.1186/1746-160X-1-9 (PMC1298321; doi:10.1186/1746-160X-1-9)
Supplement: Additional File 5 — Table 5 Crosstables for palatal measurements: preterm (LBW) vs. term (NBW). [file 1746-160X-1-9-S5.pdf]

Table 5. Crosstables for palatal measurements [mm]: preterm (LBW) vs. term (NBW) [all values are means; (SD in parenthesis)]; ~:no data given; [1] Type 1: pads meet in the region of the molar segments just as, later on, provided the occlusion is normal, mandibular molars lie lingual to the maxillary; anterior margin of the first molar segment slightly anterior to anterior margin of maxillary first molar segment. Type II: distal displacement of the mandible, anterior borders of molar segments lie posterior to anterior borders of corresponding maxillary segments. Type III: mandibular pad is entirely smaller than the maxillary (see Tab. 3, Part 1); [2]Owing to shortcomings in impressions it was not always possible to locate postginigivae with certainty [38]. * Values calculated from original data by the authors.
Due to missing information on degree of childrens´ maturity at birth no valid assignment into a group of term infants is possible with the exception of the papers [69], [74], [73] and [38]. Studies included in this table, however represent the best evidence that is possible until now. * Recalculation of original data given by [52] and [75] by the authors of the review allowed inclusion of these dissertation, while using data calculated for term or PT infants, respectively, exclusively. Data is limited to comparable measurements.

**PT / LBW**<br>– **n/ sex/**<br>– **GA at birth/ BW/**<br>– **corr. age/ weight (at impression)**<br>– **%/ hours of intubation (h)**	**Outer palatal width at lateral sulci**	**Inner palatal width at lateral sulci**	**Distance between postgingivae[2]**	**Maximum width (at the crest of the alveolar ridge perpendicular to the raphe mediana)**	**Depth with reference to the apices of the interdental papilla between primary first and second molars**	**Maximum depth (measured from a horizontal reference plane on the crest of the alveolar ridge)**	**Depths with reference to the lateral alveolar margins**
**[37]**	- 15/ 5 F, 10 M./<br>- 29 W GA (1.5)/ 1.42 kg (0.36)<br>- term/~<br>- -/-<br>22.61 (2.50)	- 15/ 5 F, 10 M/<br>- 29 W GA (1.5)/ 1.42 kg (0.36)<br>- term/~<br>- -/-<br>16.58 (1.67)	- 15/ 5F, 10 M/<br>- 29 W GA (1.5)/ 1.42 kg (0.36)<br>- term/~<br>- -/-<br>24.24 (1.66)				
**[45]**					- 52/ ~<br>- 29.4 W (3.4) / 1151 g (418.3)<br>- 2- 5 Y, ~<br>- ~/ 26 D (24.5)<br>12.9 (1.92)		
**[46]**				- 27 / ~<br>- 30.7 W (2.8) / ~<br>- 37.6 W (3.15) / ~<br>- -/ -<br>26.8 (1.54)<br>———<br>- 10 / ~<br>- 30.7 W (2.8) / ~<br>- 53.8 W (5.9) / ~<br>- -/ -<br>31.3 (2.71)		- 27 / ~<br>- 30.7 W (2.8) / ~<br>- 37.6 W (3.15) / ~<br>- -/ -<br>6.84 (0.73)<br>———<br>- 10 / ~<br>- 30.7 (2.8) W / ~<br>- 53.8 (5.9) W/ ~<br>- -/ -<br>8.83 (0.75)	
***[52]**				- *6/~<br>- ~/2132.5 g.<br>- ‘newborns’/~<br>- ~/~<br>*31.5			
**[49]**				- 23 / ~<br>- 29.2 W GA / 1215 g (227)		- 23 / ~<br>- 29.2 W GA / 1215 g (227)	

				- 29.2 W GA + 2- 7 D / ~<br>- 65 %/ 165 H (532)<br>22.6 (1.8)		- 29.2 W GA + 2- 7 D / ~<br>- 65 %/ 165 H (532)<br>6.1 (0.7)	
*[53]				- *11/ 7 F, 4 M<br>- ~/2897.8 (377.6)<br>1-7 D after birth (corrected age not given), spontaneously delivered exclusivlely, all occipito- anterior vertex presentation/~<br>- ~/ ~<br>*30.1			
[50]						- 19 / ~<br>- < 32 W (median 1880)<br>- 28 W/ ~<br>- 19 < 10 D, 4 > 10 D<br>6.2 (0.36)	- 19 / ~<br>- < 32 W (median 1880)<br>- 28 W / ~<br>- 19 < 10 D, 4 > 10 D<br>6.2 (0.36)
						- 15 / ~<br>- < 32 W/ (median 1880)<br>- 36 W./ ~<br>- 19 < 10 D, 4 > 10 D<br>7.05 (0.23)	- 15 / ~<br>- < 32 W/ (median 1880)<br>- 36 W./ ~<br>- 19 < 10 D, 4 > 10 D<br>7.05 (0.23)
						- 9 / ~<br>- < 32 W/ (median 1880)<br>- 53 W./ ~<br>- ~/ 19 < 10 D, 4 > 10 D<br>9.0 (0.15)	- 9 / ~<br>- < 32 W/ (median 1880)<br>- 53 W / ~<br>- ~/ 19 < 10 D, 4 > 10 D<br>9.0 (0.15)
						- 10 / ~<br>- < 32 W/ (median 1880)<br>- 66 W/ ~<br>- ~/ 19 < 10 D, 4 > 10 D<br>8.82 (0.76)	- 10 / ~<br>- < 32 W/ (median 1880)<br>- 66 W/ ~<br>- ~/ 19 < 10 ds., 4 > 10 D<br>8.82 (0.76)

**TERM**<br>**- n/sex/ BW/**<br>**- age / weight (at impression)**	**Outer palatal width at lateral sulci**	**Inner palatal width at lateral sulci**	**Distance between postgingivae**	**Maximum width (at the crest of the alveolar ridge perpendicular to the raphe mediana)**	**Depth with reference to the apices of the interdental papilla between primary first and second molars**	**Maximum depth (height) (measured from a horizontal reference plane on the crest of the alveolar ridge)**	**Depths < maximum depths**
[67]	- N = see below/ ~ / ~<br>- 3.7 D (2.8) (n=78)/ 3315.0 g		- N = see below/ ~ / ~<br>- 3.7 D (2.8) (n=78)/ 3315.0 g (539.5)	- N = see below/ ~/ ~<br>- 3.7 D (2.8) (n=78)/ 3315.0 g (539.5)		- N = see below/ ~ / ~<br>- 3.7 D (2.8) (n=78)/ 3315.0 g (539.5) (n=90)	

	(539.5) (n=90)<br>25.5 (1.5) (n=82)	(n=90)<br>25.5 (1.2) (n=90)	(n=90)<br>30.6 (1.5) (n=90)		7.6 (2.0)
**[68]**		- M: 89 / 3.32 kg<br>- birth / M: 3.32 kg<br>M: 25.5 (1.20)	- M: 89 / 3.32 kg<br>- birth /M: 3.32 kg<br>m.:30.6 (1.50)		- M.:89 /3.32 kg ~<br>- birth /M: 3.32 kg<br>M.:7.6 (2.0)
		- m: 36, F:26/M.: 3.32 kg, F:~<br>- 8-1 5 W/ M.: 4.28 kg, F: 3.72 kg<br>M.: 27.7 (2.36)<br>F:26.4 (1.50)	- M: 36, F: 26/ M.: 3.32 kg, F:~<br>- 8-15 W/ M.: 4.28 kg, F: 3.72 kg<br>M: 32.6 (2.68)<br>F: 30.5 (2.04)		- M: 36, F:26/M.: 3.32 kg, F:~<br>- 8-15 W/ M: 4.28 kg, F: 3.72 kg<br>M.: 9.5 (1.24)<br>F: 9.0 (1.15)
		- M: 36, F: 10/M: 3.32 kg, F:~<br>- 24-31 W/ M: 6.29 kg, F: 6.20<br>M: 30.2 (2.12),<br>F: 28.1 (1.70)	- M.:36, F: 10/M.: 3.32 kg, F:~<br>- 24- 31 W/ M.: 6.29 kg, F: 6.20<br>M.: 35.4 (2.50)<br>F: 33.6 (2.16)		- M.:36, F: 10/ M.: 3.32 kg, F:~<br>- 24- 31 W/ M.: 6.29 kg, F: 6.20<br>M.: 10.3 (1.03)<br>F: 9.6 (1.01)
**[69]**	- N = see below/ ~<br>- < 8 D / ~<br>Type I: 25.29 (n=32) [1]<br>Type II: 24.48 (n=25) [1]<br>Type III: 24.00 (n=4) [1]				- N = see below/ ~<br>- < 8 D / ~<br>Type I: 7.14 (n=32)[1]<br>Type II: 7.58 (n=25)[1]<br>Type III: 8.75 (n=4) [1]
**[70]**			- 6,/ 5 M, 1 F/ ~<br>- within one W after birth/ ~<br>34.94<br>–at six MO<br>38,38		
**[71]**			–1- 4 D after birth<br>M: 31.45 (0,08)<br>F: 30.43 (0,07)		- 1- 4 D after birth<br>M: 8.00 (0.043)<br>F: 7.81 (0,045)
**[45]**				- 45/ ~/ ~<br>- 2- 5 Y<br>11.4 (2.16).	
**[72]**				- 32/ ~/ ~<br>- ≥ 5 Y./ ~/ ~<br>13.09 (1.40)	
***[52]**			- *54/~/3469.8 g, length 50.9 cm, biggest head circumference = 35.2 cm<br>33.9		

**[74]**	- 388/ ~/ ~<br>- at birth/ ~<br>28.5 (1.45)	- 388/~/~<br>- at birth/ ~<br>20.17 (1.22)				
**[74]**	– 34 Caucasian/ ~/ 5.754 lbs (0.823)<br>– 6.5 D (3.5)/ ~<br>26.37 (1.71)<br><br>– 34 Afro– Carribean/ ~/ 5.747 (0.773)<br>– 4.9 D (2.8)<br>27.54 (1.22)<br>(p=0.002)	–34 Caucasian/ ~/ 5.754 lbs (0.823)<br>–6.5 D (3.5)/~<br>18.75 (1.49)<br><br>–34 Afro– Carribean/ ~/ 5.747 (0.773)<br>–4.9 D (2.8)<br>19.56 (1.37)<br>(p=0.024)			–34 Caucasian/ ~/ 5.754 lbs (0.823)<br>–6.5 D (3.5)/ ~<br>7.57 (0.73)<br><br>–34 Afro– Carribean/ ~/ 5.747 (0.773)<br>–4.9 D (2.8)<br>7.25 (0.78)<br>(NS)	
**[38]**		- 109 /~/~<br>- at the visit nearest 6 MO 6.5 (1.26) / ~<br>22.66 (1.713)	- 91 / ~/ ~<br>- at the visit nearest 6 MO 6.5 (1.26) / ~<br>30.91 (2.21)	- 109 / ~/~<br>- at the visit nearest 6 MO 6.5 (1.26) / ~<br>32.95 (2.46)		
***[53]**				–F: 49/ F/ 3397.4 g.<br>–1-7 D/ ~/ mean size 49.9 cm, spontaneous delivery, occipito anterior vertex presentation<br>*30.2<br>–*M:52/ M/ 3510.0 g<br>– 1-7 D/ ~/ spontaneous delivery, occipito-anterior vertex presentation<br>* 30.3		
**[75]**				–4-48 H after birth<br>M (n=525): 30.66 (0.07)<br>F (n= 470): 29.68 (0.08)		- 4- 48 H after birth<br>M (523): 8.31 (0.08)<br>F: 8.16 (0,08)
**[76]**				- at birth [71]:<br>30.99<br>- 4 MO: 33.6<br>- 8 MO: 36.12<br>- 12 MO: 37.41		- 4 MO: 10.02<br>- 8 MO: 11.31<br>- 12 MO 12.25
**[77]**				- 12/ 6 M., 6 F/ ~<br>- 0 ≤ 3 MO / ~<br>31.23		- 12/ 6 M., 6 F/ ~<br>- 0 ≤ 3 MO / ~<br>8.76
				- 9 / 4 M., 5 F/ ~<br>- 3 ≤ 6 MO / ~		- 9 / 4 M., 5 F/ ~<br>- 3 ≤ 6 MO / ~

		32.31<br>- 5/ 4 M., 1 F<br>- 6 ≤ 9 MO/ ~<br>33.65	9.61<br>- 5/ 4 M., 1 F<br>- 6 ≤ 9 MO/ ~<br>9.77
**[78]**	- 39/ M/ ~<br>1-11 D/ > 3 kg<br>26.3 (1.74)<br>- 45/ F/ ~<br>1- 11 D/ > 3 kg<br>25.9 (1.37)<br>- 84/ both sexes/ ~<br>- 1- 11 D/ > 3kg<br>26.1		

BW = birthweight, D = day(s), F = female, GA = gestational age, GW = gestational weeks; H = hour(s); LBW = low birthweight, M = male, MO = month(s), NBW = normal birthweight, NS = not significant, PT = preterm, VLBW = very low birthweight, W = weeks, Y = year(s).
