# Supplementary material for: Palatal development of preterm and low birthweight infants compared to term infants – What do we know? Part 2: The palate of the preterm/low birthweight infant
Source: Head Face Med. 2005 Oct 28;1:9. doi: 10.1186/1746-160X-1-9 (PMC1298321; doi:10.1186/1746-160X-1-9)
Supplement: Additional File 6 — Table 6 Metrical studies with respect to vertical palatal dimensions of intubated PT infants (deciduous dentition). [file 1746-160X-1-9-S6.pdf]

**Table 6.** Metrical studies with respect to vertical palatal dimension of intubated PT infants (deciduous dentition).

| Study | [45] | [23] | [48] |
|---|---|---|---|
| - age (years) | 2-5 | 2-5 | 3-5 |
| - BW (g) | Ø 1151 (SD 418) (range 530-2263) | Ø 1213 (SD ne) (range 605-1500) | Ø 993 (SD 196) (range 595-1247) |
| - GA (weeks) | Ø 29.4 (SD 3.4) | ne | Ø 28.9 (2.7) |
| - intubation time (days) | Ø 26 (SD 24.5) (range 1-90) | Ø ne (SD ne) (range 3-64) | Ø 18.3 (SD 21) (range 1-99) |
| - sucking habits | ne | no | no |
| - method | measurements of casts | measurements of casts | measurements of casts |
| - control group | yes | yes | yes |
| - palatal grooving | 25 % | 0 % | 14.6 % * |
| - high palatal vault | 69 % | ne | 63 % * |
| - differences in palatal depth | yes* | ne | ns |
| - palatal depth asymmetry | ne | ne | Yes * |

* = p<.05, ns = not significant, ne = not evaluated, Ø = mean, SD = standard deviation.
