# Supplementary material for: Palatal development of preterm and low birthweight infants compared to term infants – What do we know? Part 2: The palate of the preterm/low birthweight infant
Source: Head Face Med. 2005 Oct 28;1:9. doi: 10.1186/1746-160X-1-9 (PMC1298321; doi:10.1186/1746-160X-1-9)
Supplement: Additional File 7 — Table 7 Metrical studies with respect to vertical palatal dimensions of intubated PT infants (mixed dentition). [file 1746-160X-1-9-S7.pdf]

Table 7. Metrical studies with respect to vertical palatal dimension of intubated PT infants (mixed dentition).

Study	[48]	[10]
- age (years)	7-10	8-11
- BW (g)	Ø 1044 (SD 288, range 537-1616)	Ø ne (SD ne, range 957-2040)
- GA (weeks)	Ø 29 (SD 2.6, range 22-33)	Ø ne (range 20-37)
- intubation time (days)	Ø 26 (SD 31 range 0-99)	Ø 15 (range 1-58)
- sucking habits	no	no
- method	measurements of casts	measurements of casts
- control group	yes	yes
- palatal grooving	23 % *	ne
- high palatal vault	62% *	ne
- differences in palatal depth	ns	yes (frontal region) *
- palatal depth asymmetry	yes *	ne

* = p<.05, ns = not significant, ne = not evaluated, Ø = mean, SD = standard deviation.
