# Supplementary material for: Palatal development of preterm and low birthweight infants compared to term infants – What do we know? Part 2: The palate of the preterm/low birthweight infant
Source: Head Face Med. 2005 Oct 28;1:9. doi: 10.1186/1746-160X-1-9 (PMC1298321; doi:10.1186/1746-160X-1-9)
Supplement: Additional File 8 — Table 8 Metrical studies with respect to transverse palatal dimensions of intubated PT infants (deciduous dentition). [file 1746-160X-1-9-S8.pdf]

Table 8. Metrical studies with respect to transverse palatal dimension of intubated PT infants (deciduous dentition).

Study	[45]	[23]	[48]
		**deciduous dentition**	
- age (years)	- 2-5	- 2-5	- 3-5
- BW (g)	- Ø 1151 (SD 418) (range 530-2263)	- Ø 1213 (SD ne) (range 605-1500)	- Ø 993 (SD 196) (range 595-1247)
- GA (weeks)	- Ø 29.4 (SD 3.4)	- ne	- Ø 28.9 (2.7)
- intubation time (days)	- Ø 26 (SD 24.5) (range 1-90)	- Ø ne (SD ne) (range 3-64)	- Ø 18 (SD 21) (range 1-99)
- Method	- measurements of casts	- measurements of casts	- measurements of casts
- control group	- yes	- yes	- yes
- sucking habits	- ne	- no	- no
- crossbite	- 17 % (ns)	- ne	- 22 % *
- palatal width smaller compared to control group	- ne	- ne	- yes*
- posterior palatal width asymmetry	- ne	- no	- yes *
		**mixed dentition**	
- age (years)	-	-	7-10
- BW (g)	-	-	Ø 1044 (SD 288, range 537-1616)
- GA (weeks)	-	-	Ø 29 (SD 2.6, range 22-33)
- intubation time (days)	-	-	Ø 26 (SD 31 range 0-99)
- method	-	-	measurements of casts
- control group	-	-	yes
- sucking habits	-	-	no
- crossbite	-	-	21.3 % *
- palatal width smaller compared to control group	-	-	yes *
- posterior palatal width asymmetry	-	-	yes *

* = p<.05, ns = not significant, ne = not evaluated, Ø = mean, SD = standard deviation.
