# Supplementary material for: Palatal development of preterm and low birthweight infants compared to term infants – What do we know? Part 2: The palate of the preterm/low birthweight infant
Source: Head Face Med. 2005 Oct 28;1:9. doi: 10.1186/1746-160X-1-9 (PMC1298321; doi:10.1186/1746-160X-1-9)
Supplement: Additional File 9 — Table 9 Metrical studies with respect to transverse palatal dimensions of intubated PT infants (mixed dentition). [file 1746-160X-1-9-S9.pdf]

Table 9. Metrical studies with respect to transverse palatal dimension of intubated PT infants (mixed dentition); (* = p<.05, ns = not significant, ne = not evaluated, Ø = mean, SD = standard deviation).

**Study**	**[48]**	**[10]**
- age (years)	7-10	8-11
- BW (g)	Ø 1044 (SD 288, range 537-1616)	Ø ne (SD ne, range 957-2040)
- GA (weeks)	Ø 29 (SD 2.6, range 22-33)	Ø ne (range 20-37)
- intubation time (days)	Ø 26 (SD 31 range 0-99)	Ø 15 (range1-58)
- method	measurements of casts	measurements of casts
- control group	yes	yes
- sucking habits	no	no
- crossbite	21.3 % *	ns
- palatal width smaller compared to control group	yes *	yes *
- posterior palatal width asymmetry	yes *	yes *
